# Docetaxel Loaded PEG-PLGA Nanoparticles: Optimized Drug Loading, *In*-*vitro* Cytotoxicity and *In*-*vivo* Antitumor Effect 

**Published:** 2014

**Authors:** Mona Noori Koopaei, Mohammad Reza Khoshayand, Seyed Hossein Mostafavi, Mohsen Amini, Mohammad Reza Khorramizadeh, Mahmood Jeddi Tehrani, Fatemeh Atyabi, Rassoul Dinarvand

**Affiliations:** a Novel Drug Delivery Lab, Faculty of Pharmacy, Tehran University of Medical Sciences, Tehran 1417614411, Iran.; b Nanotechnology Research Centre, Faculty of Pharmacy, Tehran University of Medical Sciences, Tehran, Iran.; c Department of Drug and Food Control, Faculty of Pharmacy and Pharmaceutical Quality Assurance Research Center , Tehran University of Medical Sciences, Tehran, Iran.; d Department of Medicinal Chemistry, Faculty of Pharmacy, Tehran University of Medical Sciences, Tehran, Iran.; e Department of Medical Biotechnology, School of Advanced Medical Technologies, Tehran University of Medical Sciences, Tehran, Iran.; f Monoclonal Antibody Research Center, Avicenna Research Institute, ACECR, Tehran, Iran.

**Keywords:** PLGA, Nanoparticles, Docetaxel, Anti-tumor, Pegylation, Experimental design, Box-Behnken

## Abstract

In this study a 3-factor, 3-level Box-Behnken design was used to prepare optimized docetaxel (DTX) loaded pegylated poly lactide-co-glycolide (PEG-PLGA) Nanoparticles (NPs) with polymer concentration (X_1_), drug concentration (X_2_) and ratio of the organic to aqueous solvent (X_3_) as the independent variables and particle size (Y_1_), poly dispersity index (PDI) (Y_2_) and drug loading (Y_3_) as the responses. The cytotoxicity of optimized DTX loaded PEG-PLGA NPs was studied in SKOV3 tumor cell lines by standard MTT assay. The *in-vivo *antitumor efficacy of DTX loaded PLGA-PEG NPs was assessed in tumor bearing female BALB/c mice. The optimum level of Y_1_, Y_2_ and Y_3_ predicted by the model were 188 nm, 0.16 and 9% respectively with perfect agreement with the experimental data. The *in-vitro *release profile of optimum formulation showed a burst release of approximately 20% (w/w) followed by a sustained release profile of the loaded drug over 288 h. The DTX loaded optimized nanoparticles showed a greater cytotoxicity against SKOV3 cancer cells than free DTX. Enhanced tumor-suppression effects were achieved with DTX-loaded PEG-PLGA NPs. These results demonstrated that optimized NPs could be a potentially useful delivery system for DTX as an anticancer agent.

## Introduction

Nanoparticles (NPs) are colloidal particles having a size at nanometer range less than 1000 nm (1). Their higher ratio of surface area to volume provides for their improved pharmacokinetics and biodistribution of therapeutic agents and thus minimizing toxicity by their special accumulation at the target site. They also improve the solubility of hydrophobic compounds and make them suitable for parenteral administration. Furthermore, they amplify the stability of a range of sensitive therapeutic agents, such as peptides, oligonucleotides, and proteins (2). 

Encapsulation of cytotoxic chemotherapeutic agents in biodegradable polymers offers many advantages. Poly d,l-lactide-co-glycolide (PLGA) has been widely used for the preparation of NPs carrying cytotoxic drug agents (3). Hydrophilic and hydrophobic agents can be encapsulated in PLGA NPs. The drug release rates from PLGA NPsan be modified to specific applications. Their size and drug loading are very restricting to give more control over drug delivery (4).

Perfectly, a nanocarrier should be able to provide extended blood circulation, delivering the active moiety at the targeted site. Nanocarriers are quickly removed by reticuloendothelial system (RES) from the circulation, hence limiting their ability to reach target cells and consequently limiting their effectiveness. The liver Kupffer cells and macrophages in the spleen segregate the encapsulated cytotoxic chemotherapeutic agents after the NPs’ surface are opsonized with proteins. This segregation is facilitated by the recognition of the substrate by opsonization of nanocarriers’ surface by proteins (5). This opsonization process can be hindered by incorporating hydrophilic agents like PEG, on the surface of the NPs. This confers a stealth hydrophilic barrier which delays opsonization and, in turn, delays rapid recognition by the RES, leading to enhance circulation time (6, 7).

Other researchers have studied pegylated PLGA NPs (8, 9). The huge interest in these systems, however, does not eliminate the need for further works especially in the area of formulation optimization of PLGA NPs to further enhance their application as targeted drug delivery systems.

Docetaxel (DTX), a toxoid, is effective as a microtubule depolymerization agent. It has been shown to be highly effective against breast, pancreatic, gastric and urothelial carcinomas (10, 11).

In preparation of NPs by nanoprecipitation method, there are many factors that influence the outcome (12). Experimental design has many advantages such as reducing the number of experiments and hence saving time and money. To carry out experimental design, a mathematical model to assess the significance and statistical meaning of the factor effects and estimate of interaction effect between considered factors is to be developed (13, 14). This useful information cannot be obtained by “one factor at a time” studies (15). Therefore, the subsequent desirability function followed by response surface methodology permits evaluation of the best model to find the optimal solution for the system (16).

The purpose of this study was to produce high quality pegylated PLGA NPs containing DTX, *i.e*. with minimum size, maximum drug loading and minimum poly dispersity. The *in-vitro *release profile and both *in-vitro *and *in-vivo *cytotoxicity effect of optimum formulation confirmed the desirable efficacy of DTX loaded pegylated PLGA NPs.

## Experimental

*Materials*

PLGA (50:50, Resomer RG 504H, MW 48000) was purchased from Boehringer Ingelheim (Ingelheim, Germany). Poly vinyl alcohol (PVA) (MW 22000) and bifunctional NH2-PEG-OH (weight average MW 5000) were purchased from Sigma-Aldrich (St. Louis, Missouri, USA). N-hydroxysuccsuccinimide (NHS), dicyclohexylcarbodiimide (DCC), N,N-diisopropylethylamine (NIPEA), acetone, methanol, acetonitrile and dicholoromethane (analytical grade) were purchased from Merck (Darmstadt, Germany). DTX active pharmaceutical powder was purchased from Cipla Pharmaceutical Co. (Mumbai, India). Deionized water was used throughout the experiment. All other chemicals used were of reagent grade. 

The breast cancer cell lines, SKOV3 (American Type Culture Collection) were obtained from the Pasteur Institute (Tehran, Iran). The cell lines were cultivated in RPMI-1640 medium supplemented with 10% fetal bovine serum and 1% penicillin streptomycin at 37 °C in a humidified incubator with 5% CO2. The 4T1 mammary carcinoma cell line was provided by Pasteur Institute of Iran, Tehran, Iran. 4T1 cells were maintained in RPMI-1640 Medium (American Type Culture Collection [ATCC], Manassas, VA), 10% FBS, 1% Glutamax-1, 1% sodium bicarbonate, 3-(4,5-dimethylthiazol-2-yl)-(2,5-diphenyl tetrazolium bromide) (MTT).

Female BALB/c 6 to 8 weeks of age, were purchased from Pasteur Institute of Iran, Tehran, Iran. All animal experiments were performed according to National Laboratory Animal Facilities guidelines. 4T1 Cell lines were grown in 37 °C, 5% CO2 incubators. 

*Methods*

*Synthesis of PLGA–b–PEG*

The conjugation of OH–PEG–NH_2_ to PLGA–COOH was carried out via an activation of carboxylic acid by NHS and EDC(8). PLGA-COOH (0.28 mmol) was converted to PLGA-NHS in methylene chloride (10 mL) with excess of NHS (135 mg, 1.1 mmol) in the company of EDC (230 mg, 1.2 mmol). PLGA-NHS was precipitated with ethyl ether (5 mL), and repeatedly washed in an ice-cold mixture of ethyl ether and methanol to take out residual NHS. After drying under vacuum, PLGA-NHS (1 g, 0.059 mmol) was dissolved in chloroform (4 mL) followed by addition of OH-PEG-NH_2_ (250 mg, 0.074 mmol) and NIPEA (28 mg, 0.22 mmol). The co-polymer was precipitated with cold methanol after 12 h and washed with the same solvent (35 mL) to take out unreacted PEG. The resulting PLGA–PEG block co-polymer was dried under vacuum and used for NP preparation without further treatment. The NMR peaks of the copolymer are as follows:

^1^H-NMR (CDCl3 at 300Hz) δ 5.2 (m, ((OCH(CH3)C(O)OCH2C(O))n-(CH2CH2O)m), 4.8 (m,((OCH(CH3)C(O)OCH2C(O))n-(CH2CH2O)m), 3.7 (s, ((OCH(CH3)C(O)OCH2C(O))n-

(CH2CH2O)m), 1.6 (d, ((OCH(CH3)C(O)OCH2C(O))n-(CH2CH2O)m).

*Preparation of DTX-loaded PLGA–b–PEG NPs*

The nanoprecipitation method was employed for the preparation of pegylated PLGA NPs containing DTX. Briefly, DTX and PLGA-b-PEG were dissolved in acetone as an organic solvent that is miscible with water. The drug-polymer solution was then added to an aqueous phase containing 0.5% PVA (W/V). The mixture was probe sonicated at 5 amplitudes for 60 seconds to produce an O/W emulsion. The O/W emulsion was stirred using a magnetic stirrer for 4 hours at room temperature until the evaporation of the organic phase was completed to generate NPs. The NPs were then collected by centrifugation for 15 min at 10,000 g. The PLGA–b–PEG NPs were re-suspended, washed with water, and collected similarly (17).

*NPs characterization*

Freeze-dried NPs were dispersed in deionized water (pH=7.0) at a concentration of approximately 1 mg/mL. Dynamic light-scattering detector (Zetasizer Nano ZS 3000HS, Malvern, UK) was used to analyzed Average size and zeta potential of nanoparticle at 25 °C and at scattering angle of 90°.The particle size and zeta potential of each sample was determined three times and the average values were calculated. 

The shape and surface morphology of the produced NPs was observed by scanning electron microscopy (SEM, Philips XL 30 scanning microscope, Philips, the Netherlands) was NPs were coated with gold under vacuum before SEM.

*Experimental design*

Our preliminary experiments and other studies have indicated that the variables mostly affecting the preparation of PLGA NPs by nanoprecipitation technique were the amounts of polymer, concentration of the drug, the ratio of organic to aqueous phase. Box-Behnken design was particularly used to statistically optimize the formulation parameters and evaluate the main interaction effects since it requires a small number of runs in case of three or four variables. A 3-factor, 3-level Box-Behnken design was used to optimize NP formulation with polymer concentration (*X*_1_), drug concentration (*X*_2_) and the ratio of organic to aqueous solvent (*X*_3_) as the independent variables with low, medium and high concentration values presented in Table 1. 

The Box-Behnken designs 17 experiments, including 12 factorial points, with 5 replicates at the center point for estimation of pure error sum of squares, were employed. Range of concentrations was recognized according to earlier studies for development of NPs. The dependent variables were particle size (*Y*_1_), poly dispersity index (*Y*_2_) and drug loading (*Y*_3_) applied as described in Table 1. Design-Expert^®^ software (v.7.1.5 Stat-Ease *Inc*., Minneapolis, USA) was used for the creation and evaluation of the statistical experimental design. The concentrations of the formulation parameters and the corresponding observations for these dependent variables are presented in Table 2. A second-order polynomial function as follows can model the mathematical relationship between the dependent variables (*Y*) and the independent variables (*X*_i_) (18):

Equation (1) $$Y=\beta_{0}+\beta_{1}X_{1}+\beta_{2}X_{2}+\beta_{3}X_{3}+\beta_{11}X_{1}2+\beta_{22}X_{2}2+\beta_{33}X_{3}2+\beta_{12}X_{1}X_{2}+\beta_{13}X_{1}X_{3}+\beta_{23}X_{2}X_{3}$$

Where *Y *is the predicted response; *β*_◦_, intercept; *β*_1_, *β*_2_ and *β*_3_, linear coefficients; *β*_11_*, β*_22_ and *β*_33_, squared coefficients; *β*_12_, *β*_13_ and *β*_23_, the interaction coefficients of the equation; *X*_1_, *X*_2 _and X_3_*, *the independent variables. Using this equation, it is possible to evaluate the linear, quadratic and interactive effects of the independent variables on the response.

For the purpose of nanoparticle formulation optimization, three-dimensional response surface plots from the experimental data were drawn. All responses observed were fitted to linear, second order and quadratic models, and were evaluated in expressions of statistically significant coefficients p-values and *R*^2 ^values. Polynomial equations relating the major effect and interface factors were determined based on evaluation of statistical parameters such as multiple correlation coefficient, adjusted multiple correlation coefficient and the predicted residual sum of squares generated by Design-Expert software. The statistical corroboration of the polynomial equations was established by ANOVA through Fisher’s test and shown by a p value below 0.05, which is provision available in the software. Therefore, the optimum values of the variables were obtained by graphical and numerical analyses using the Design-Expert software and based on the criterion of desirability (19). In order to graphically show the relationship and interactions between the coded variables and the response, contour plots and three-dimensional surface plots were used in this study. The optimal points were determined by solving the equation derived from the final quadratic model and grid search of RSM plot. NPs were organized using the optimal formulation, and resultant experimental values of the responses were quantitatively compared with the predicted values for calculating the percentage of the predicted error. Predicted error is the variation between the experimental value and the predicted value per predicted value (20, 21). Validation of the optimization procedure was demonstrated for predicted errors lower than 5% (20). 

*Optimization by desirability function*

Desirability function was used, for the concurrent determination of the optimum setting of input variable that can determine optimum performance levels for one or more responses. The initial desirability function was developed by Harrington (22). Later Derringer specified the relationship between predicted responses on dependent variables and the desirability of the responses (23). The desirability method consists of two steps: First, finding the desirable input factor that lead to the most desirable predicted response on the responses and second, maximizing the overall desirability with appreciating the controllable factors. Therefore the desirability function by the simple and quick transformation of different responses to one measurement can be employed to obtain qualitative and quantitative responses.

Dirringer’s desirability function, D, is defined as geometric mean, waited, or otherwise, of individual desirability function (23). The equation that defines the Dirringer’s desirability function is:

Equation (2) $$D={\left[d_{1}p^{1}.d_{2}p^{2}.d_{3}p^{3}\ldots \ldots \mathrm{..}d_{n}p^{n}\right]}^{1/n}$$

Where d_i_ indicates the desirability of the response, n is number responses in the measure and p_i_ is the weight of the response. Weight can range from 0.1 to 10. Weights greater than 1 gives more emphasis to goal whereas lower than 1 gives less emphasis. d_i_ is converted response, that varies from 0 to 1 (d_i_=0,for a completely undesired response and d_i_=1 for a fully desired response). If any of the responses beyond the desirability, then overall function turned in to zero:

Equation (3) D=1/n,0

Maximum overall desirability function D, depends on the importance value. For simultaneous optimization each response must have a low and high value assigned to each goal. Therefore ‟in range” are included in the product of the desirability function ‟D”, but are not counted in determining ‟n”: D= 1/n. If the goal is none, the response will not be used for the optimization.

In this study the ANOVA results for relationship between the responses and independent variables are presented in Table 3.

*Drug loading and release study*

Lyophilized NPs (2.5 mg) were dissolved in 1 mL of acetonitrile and shaken lightly followed by sonication for 6 min. Then, 2 mL of methanol was added to precipitate the polymer. The sample was filtered and drug quantity in filtrate was determined by HPLC analysis. The drug loading was determined as the relative amount of drug content of NPs to the whole weight of the NPs (24). 

HPLC analysis was performed at 35 °C, using a Knauer apparatus (model K-1001, WellChrom, Berlin, Germany) equipped with a reversed-phase C18 column (25 cm × 0.46 cm internal diameter, pore size 5 μm; Teknokroma, Barcelona, Spain) and eluted isocratically with acetonitrile/water (65/35 v/v). The flow rate was fixed at 1 mL/min and detection was obtained by UV detection at 230 nm. The linear regression coefficient determined in the range 0.05–10 μg/mL was 0.9994 (n=6). The method sensitivity was 0.05 μg/mL with signal to noise ratio of 3:1.

2.5 mg of freeze-dried DTX-loaded NPs suspended in 10 mL of isotonic pH 7.4 phosphate buffer saline solution (PBS), were poured in a dialysis bag. Then the dialysis bag was placed in 50 mL of PBS. The whole assembly was maintained at 37 ± 0.5 °C, covered by parafilm to avoid evaporation and shaken at 90 cycles/min. At fixed time intervals, 2 mL of medium were withdrawn and replaced with the same volume of fresh buffer to maintain the required sink condition. This was taken into account while calculating cumulative drug release. The sample was filtered and drug quantity in filtrate was determined by HPLC analysis. Quantification was done by calibration curve of DTX in respective buffer solution.

*In-vitro cytotoxicity of DTX-loaded NPs*

The cytotoxicity of optimized NPs was studied in SKOV-3 cells using the MTT assay (25). Briefly, SKOV-3 cells were seeded in 96-well plates (Costar, Chicago, IL) at the density of 1 × 10^4^ viable cells/well and incubated for 24 hours to allow cell attachment. The medium was replaced by 100 μL of the formulation at concentrations of 1–150 nM for 24 hours. For free docetaxel, a stock solution was prepared in dimethyl sulfoxide (1 mg/mL docetaxel). The dimethyl sulfoxide concentration in the medium was lower than 0.5%, at which level it has no effect on cell proliferation. The diluents for preparing the working solution for free docetaxel drug and NPs was RPMI-1640 culture medium. At designated time intervals, 20 μL MTT (5 mg/mL in phosphate-buffered saline) was added to each well, and the culture medium containing MTT solution was removed after 3–4 hours. The formazan crystals were dissolved in 100 μL dimethyl sulfoxide and read at 570 nm by a microplate reader. Cell viability was calculated using the following equation:

Equation (4) $$\mathrm{Cell Viability}\left(\%\right)=({\mathrm{Int}}_{s}/{\mathrm{Int}}_{\mathrm{control}})\times 100$$

Where Int_s_ is the colorimetric intensity of cells incubated with the samples, and Int_control _is the colorimetric intensity of cells incubated with the phosphate-buffered saline only (positive control).

*Antitumor efficacy of DTX loaded optimized PLGA-PEG NPs in tumor bearing mice*

The *in-vivo *antitumor efficacy of DTX loaded optimized PLGA-PEG NPs was assessed in female BALB/c mice (body weight = 20 to 25 g). 4T1 tumors were induced in BALB/c mice by subcutaneous injection of about 10^6^ cells dispersed in PBS on the dorsal side. Tumors were measured using Vernier calipers every alternate day. When tumors reached a volume of 150 mm^3^, samples were injected through the tail vein. Animals were randomly divided into four groups (control, Taxotere®, plain NPs, and DTX loaded NPs), with each group having six mice. Mice were treated with a single IV injection of 10 mg/Kg body weight dose equivalent to DTX in each group. The control group mice received a single IV injection of saline. At predetermined time intervals, tumor volume was determined by measuring its dimensions using digital calipers and calculated according to the following formula (26) . 

Equation (5) Tumor volume (mm^3)=0/52(long Diameter × Short Diameter)2

Throughout the experiments, all animals were accommodated in a conventional pathogen-free laboratory environment. The study was terminated at 17 days post treatments.

*Statistical analysis*

One-way analyses of variance were performed for comparison of the results. p-values of 0.05 were considered to be statistically significant.

## Results and Discussion

**Figure 1:**
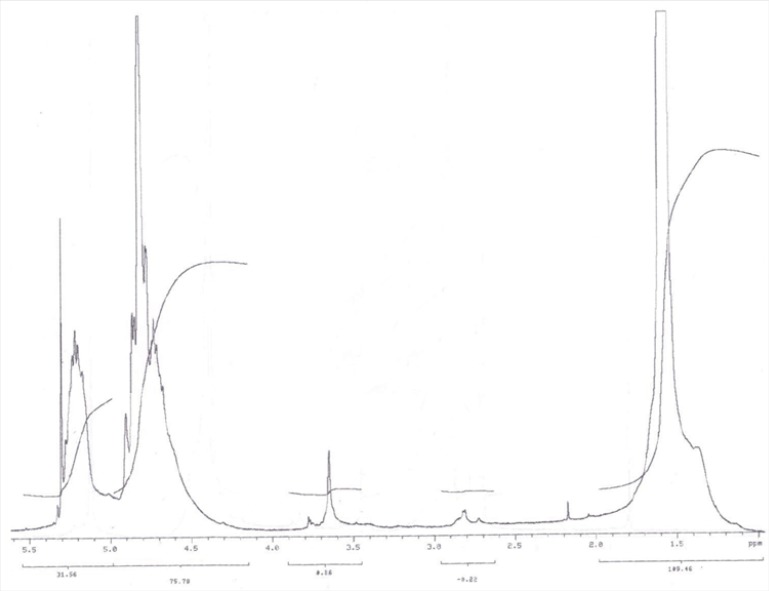
^1^H NMR spectrum of PEG–PLGA copolymer

PLGA-PEG-OH was synthesized by direct conjugation of PLGA-COOH and NH_2_-PEG-OH. The basic chemical structure of PEG–PLGA copolymer is confirmed by H NMR in Figure 1.

One of the striking features is a large peak at 3.65 ppm, corresponding to the methylene groups of the PEG. Overlapping doublets at 1.55 ppm are attributed to the methyl groups of the D- and L-lactic acid repeat units. The multiples at 5.2 and 4.8 ppm correspond to the lactic acid CH and the glycolic acid CH, respectively, with the high complexity of the peaks resulting from different D-lactic, L-lactic glycolic acid sequences in the polymer backbone. Hydroxyl group located at the end terminal of hydrophilic PEG block is available for surface chemistry on the nanoparticle surface. The efficiency of coupling reaction was determined by H^1^ NMR witch revealed that approximately 70% of PLGA conjugated with PEG.

**Table 1:** Variables in Box–Behnken design

**Factor**	**Levels used**		
**Independent variables**	**−1**	**0**	**1 **
*X*_1_ = polymer concentration (mg/mL)	7	11	15
*X*_2_ = drug concentration (mg/mL)	0.2	1.1	2.0
*X*_3_ = ratio of solvent to water	0.1	0.3	0.5
**Dependent variables**		**Constraints **	
*Y*_1_ = Particle size (nm)		Minimize	
*Y*_2 _= Polydispersity index		Minimize	
*Y*_3_ = loading (%)		Maximize	

*Effect of preparation variable on formulation characteristics*

In this work, the variables such as polymer concentration, drug concentration and ratio of the solvent to water, during the nanoparticle preparation were studied. A technique of Box-Behnken experimental design which fits a full quadratic second-order polynomial equation to the data, offers the possibility of investigating three independent factors at two levels after performing seventeen experiments. Preliminary works carried out to select the factors and their level in the study, which affected size, polydispersity and loading efficiency.

**Figure 2:**
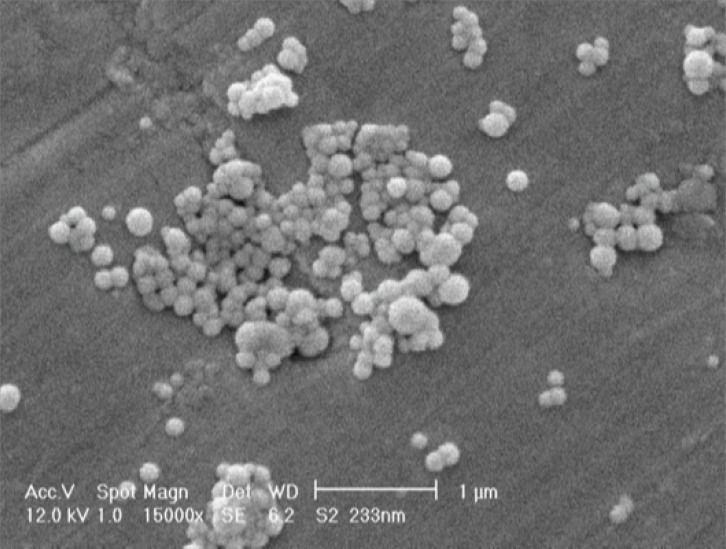
Scanning electron micrographs of docetaxel loaded PEG-PLGA NPs

As shown in Table 2, the size of NPs ranged from 186 to 267 nm for different experiments. The zeta potential of the NPs was negative due to their coating with free hydroxyl group of the PEG. Figure 2 shows the SEM photograph of the spherical NPs. Box-Behnken design response surface methodology for optimization.

**Table 2:** Composition and observed responses in Box–Behnken design

**Item**	**Dependent variables**				**Independent variables**		
	**X1**	**X2**	**X3**	**Y1**		**Y2**	**Y3 **
	Polymer concentration (mg/ml)	Drug concentration (mg/ml)	Ratio of solvent to water	Size		PDI	% loading
1	0.3	0.2	7		183	0.21	0.17
2	0.3	0.2	15		198	0.07	0.15
3	0.3	2.0	7		267	0.47	11.54
4	0.3	2.0	15		222	0.24	3.70
5	0.1	1.1	7		185	0.37	2.00
6	0.1	1.1	15		200	0.28	1.46
7	0.5	1.1	7		168	0.13	5.39
8	0.5	1.1	15		218	0.07	1.15
9	0.1	0.2	11		202	0.06	0.14
10	0.1	2.0	11		206	0.32	5.23
11	0.5	0.2	11		201	0.14	0.40
12	0.5	2.0	11		207	0.14	5.04
13	0.3	1.1	11		218	0.37	9.03
14	0.3	1.1	11		226	0.38	8.20
15	0.3	1.1	11		243	0.26	7.71
16	0.3	1.1	11		185	0.31	8.56
17	0.3	1.1	11		184	0.12	7.22

*Box-Behnken design response surface methodology for optimization*

The Box-Behnken design followed by response surface methodology was applied to optimize the levels of the independent variables. In this work, the important factors in formulation were analyzed and optimized by Design-Expert. In Table 2, Polymer concentration, Drug concentration, and ratio of solvent to water as the independent variables and particle size, PDI and loading % as the responses are shown. Analysis of variance (ANOVA) was used to choose the best model fitted to the data. As a result, a quadratic model was fitted to the data obtained for particle size and drug loading while for PDI the selected model was linear. In addition, the lack of fit F-value of these models was not significant. 

The multiple correlation coefficient (*R*^2^) and adjusted *R*^2^ of the model predicting the particle size were 0.999 and 0.998, respectively. This means that the model can explain more than 99.9% of the variability in the response and that only less than 0.01% of the variability is due to noise. In addition, the similarity between the *R*^2^ and adjusted *R*^2^ shows the adequacy of the model to predict the response in the optimization process. These values for the drug loading and PDI are 0.949 and 0.910, 0.965 and 0.921, respectively.

The multiple regression analysis by the software indicated the following models for the particle size, loading and PDI:

Equation (6) $$\mathrm{Particle size}=\mathrm{203.25}+16.44X_{1}+2.69X_{2}+0.12X_{3}-39.12X_{1}X_{2}+\mathrm{.7}X_{1}X_{3}+\mathrm{o.5}X_{2}X_{3}+13.56{X_{1}}^{2}+\mathrm{24.81}{X_{2}}^{2}-24.06{X_{3}}^{2}$$

From the equation 6 it is obvious that the main effect of *X*_1_ (polymer concentration), *X*_2_ (drug concentration) and *X*_3_ (ratio solvent to water) had significant positive effect on the particle size of NPs. If these factors are increased then particle size may be increased. Our results are in good agreement with other works reported the effect of solid concentration in controlling the size of NPs (27). The final size of NPs depended on the net shear stresses of the sonication for breakdown of the droplets. With increasing the solid concentration in the organic phase, the viscosity increased. The viscosity force is against the shear stresses in the organic phase. The interaction effects of polymer concentration and drug concentration (*X*_1_*X*_2_) have significant negative interaction on particle size of NPs.

Figure 3A shows the 3D response surface plotted by Design Expert software. In each plot the interaction of two variables was investigated at the same time while the third one was in its middle level value. The curvature in both variables is considerable at opposite direction. The concave shape of the plot indicates that the optimum value (minimum value) for the particle size is in the range of variable studied. As can be predicted from the positive sign of *X*_1_^2^ and *X*_2_^2^ in equation 3 as shown in Figure 3A, the minimum level of particle size were measured near the low levels of both the polymer and drug concentrations.

**Figure 3A:**
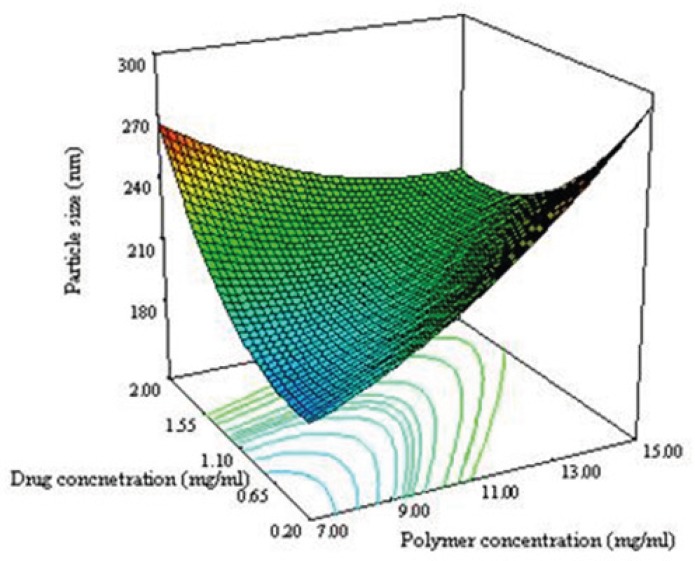
Response surface plot illustrating the enhancement of polymer concentration and drug concentration on particle size

Figure 3B shows that the ratio and polymer concentration affected simultaneously the particle size while the drug concentration was in its middle level value. The size of NPs decreased from 230 nm to 184 nm with decreasing the polymer concentration from 15 to 7 mg/mL. It has been reported that the size of NPs for a fixed polymer concentration, remain relatively unchanged when the ratio was in the range of 0.1-0.5 (8). The same effect was seen in this study as shown in Figure 3B. No significant difference in particle size between 0.1 and 0.5 of drug polymer ratio was observed. Maximum particle size of NPs was observed for the drug polymer ratio of 0.3. 

Equation (7) $$\mathrm{Loading}(\%)=\mathrm{8.14}-1.58X_{1}+3.08X_{2}+0.39X_{3}-1.95X_{1}X_{2}—2.23{X_{1}}^{2}-2.02{X_{3}}^{2}-3.42{X_{3}}^{2}$$

As seen in equation 7, the effect of variables on the loading efficacy and the interaction between them are significant. Significant interaction between the variables indicate that, the responses close to specific variables will depend on the level of other variables providing for the selection of a multilevel factorial design for study of the effect of variable on the loading efficacy of NPs.

**Figure 3B:**
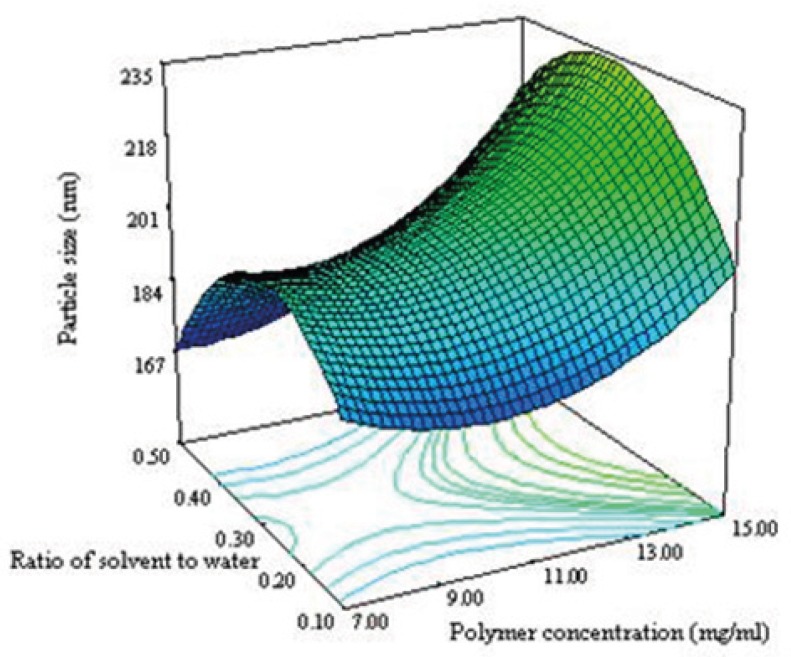
Response surface plot illustrating the enhancement of polymer concentration and ratio of solvent to water on particle size

The negative sign of X_1_^2^ and X_2_^2^ in equation 6 indicated that the optimum value (maximum value) for response in the range of variables can be found. The convex shape of the plot shown in Figure 3C supported this consideration. The drug loading increases when the drug concentration and ratio of solvent to water are increased. During the emulsification process, DTX was dissolved in organic phase, so when the ratio of solvent to water increases, the amount of the drug increases as well leading to an increase in the particle size of NPs. By increasing the particle size, the length of the diffusion path way in to the aqueous phase increases, thereby reducing the drug loss through diffusion and increasing drug content (28). 

Equation (8) $$\mathrm{PDI}=\mathrm{0.23}-0.065X_{1}+\mathrm{0.087}X_{2}-0.069X_{3}$$

It can be seen from equation 7 that drug concentration (*X*_2_) have negative effect PDI. This may be due to greater viscosity by increasing the solid concentration explained above (27). 

By solving equations 4, 5 and 6, desirability function and analyzing the response of surface plots obtained by Design Expert software, we found that the optimum values of independent variables in uncoded (actual) units to optimize the responses are a polymer concentration of 9.75 mg/mL, drug concentration of 1.25 mg/mL and solvent to water ratio of 0.31. Under these conditions, the optimum level of size, PDI and drug loading predicted by the model were calculated to be 188 nm, 0.16 and 9 % respectively.

**Figure 3C:**
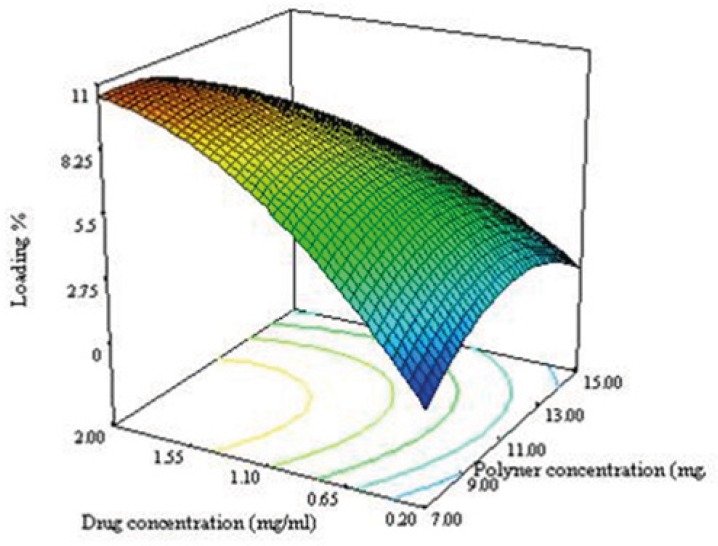
Response surface plot illustrating the enhancement of polymer concentration and drug concentration on drug loading.

For validation of the model, three experiments were performed by using the optimum condition as mentioned previously to find the particle size, drug loading and PDI. 188, 9 and 0.22 were obtained using this optimum condition; these are about 93%, 85% and 91% of the value predicted by the model respectively. The perfect agreement between the observed values and the values predicted by the equation confirms the statistical significance of the model and its adequate precision in predicting the optimum condition in the domain of levels chosen for the independent variables.

**Table 3:** Sequential model sum of square for relationship between the responses and independent variables

**p-value**	**f-value**	**Mean squares**		**df**		**Sum of squares**		**Source**				
0.0187	1552.500	776.250		3		2328.750		Quadratic vs 2FI		Y1= Particle size	
0.0199	4.675	0.044		3		0.132		Linear vs Mean		Y2= PDI	
0.0002	29.469	32.259		3		96.778		Quadratic vs 2FI		Y3= Loading %	
**Lack of fit test**												
0.6487	1552.500		776.250		3		2328.750		Quadratic vs 2FI		Y1= Particle size
0.6300	0.824		0.009		9		0.080		Linear vs Mean		Y2= PDI
0.1166	3.762		1.886		3		5.658		Quadratic vs 2FI		Y3= Loading %

*In-vitro drug release*

**Figure 4:**
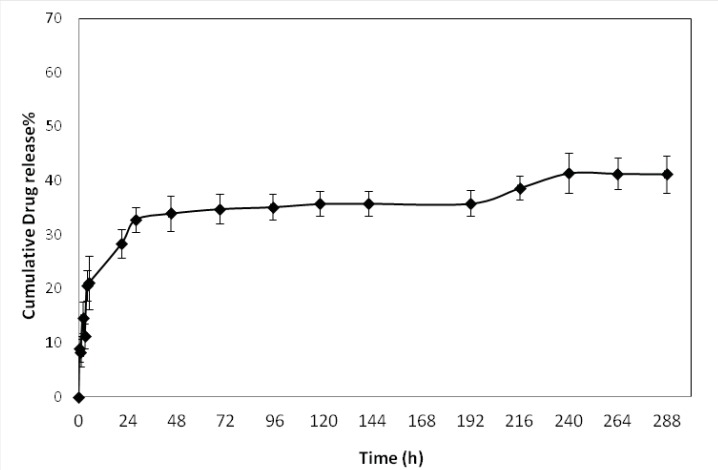
*In-vitro *drug release profile of docetaxel loaded PLGA-b-PEG NPs produced by solvent evaporation methods, in phosphate buffer saline solution (pH 7.4). Data represent mean ± SD.

The *in-vitro *release profile of optimized formulations is shown in Figure 4. The release behavior of DTX from the NPs exhibited a biphasic pattern, it consists of an initial burst during the first day (approximately 33%), followed by slower sustained release. The initial burst release of drug could be explained by diffusion of drug molecules on the surface of the NPs. This initial release was followed by more controlled release for 12 days of release time.

*In-vitro cytotoxicity of DTX-loaded NPs*

**Figure 5:**
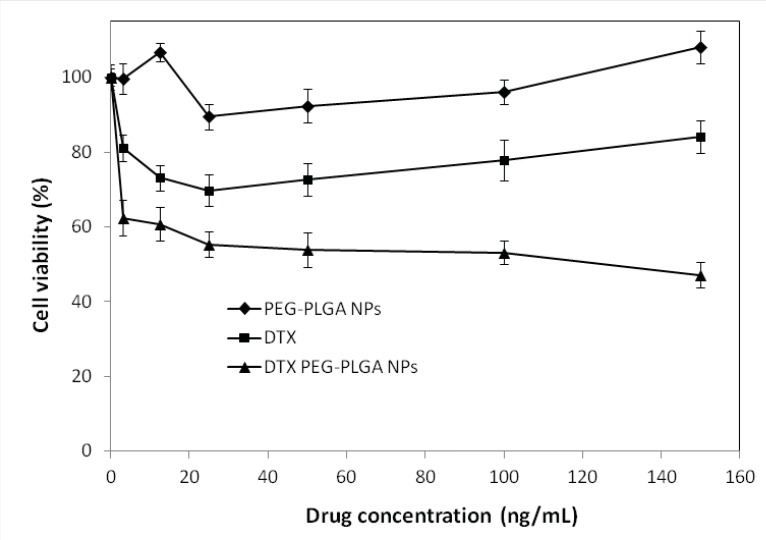
Viability of SKOV3 cells overexpressing HER2, with docetaxel formulations after 24 hours. Different concentrations of docetaxel ranging from 3-150 ng/mL either as solution (free docetaxel) or loaded in NPs (NP-DTX) were tested. NPs without any drug loading were used as controls.

The *in-vitro *cytotoxic effect of DTX free drug and DTX–loaded optimized NPs for SKOV3 cells (n=6) is shown in Figure 5. DTX loaded pegylated PLGA NPs showed significant dose-dependent cytotoxicity against SKOV3 cells. The results also demonstrate that the pegylated PLGA NPs had more cytotoxic effect than the free drug for SKOV3 cells at most concentrations. That may be due to the P-glycoprotein activity pumping out the DTX entered the cells while PLGA NPs are taken up by cells through endocytosis, hence escaping the P-glycoprotein pumps (29), which is in conformity with other works (30-32). Therefore, NPs perform as intracellular drug depots, slowly releasing the encapsulated drug into the cellular cytoplasm. In addition, our study indicated that free NPs had no effects on the cell viability (P > 0.5). Because the composition of NPs was a good biocompatible material, could be totally metabolized and non-toxic to cells.

*In-vivo anti-tumor study*

To demonstrate the antitumor efficacy, Taxotere®, NPs with no drug, and DTX loaded PEG-PLGA NPs were injected in 4T1 human breast cancer-bearing mice via lateral tail vein. The dose of DTX was 10 mg/Kg. As depicted in Figure 6A, Tumor size was measured in three and five days after injection of the samples with no significant differences observed among any of the treatment groups. However, the tumor size increased significantly in the Normal Saline (316%), the NPs without drug (365%), Taxotere® (216%), and DTX loaded PEG-PLGA NPs (126%) groups after 9 days in comparison with the initial tumor size. The DTX-NPs were more effective than Taxotere® in controlling the growth of 4T1 tumors. One *i.v*. dose of the DTX-NPs inhibited the growth of the 4T1 tumors for nine days after injection, whereas the same molar dose of Taxotere® did not significantly affect the growth of the 4T1 tumors (p = 0.219 on day 9, control vs. Taxotere®). This longer time antitumor efficacy of DTX loaded PEG-PLGA NPs resulted from their high tumor targeting ability characteristics, and sustained release profile of DTX from NPs within the tumor tissue. 9 days after injection day tumors volume increase because drug omitted from animals’ body.

**Figure 6A:**
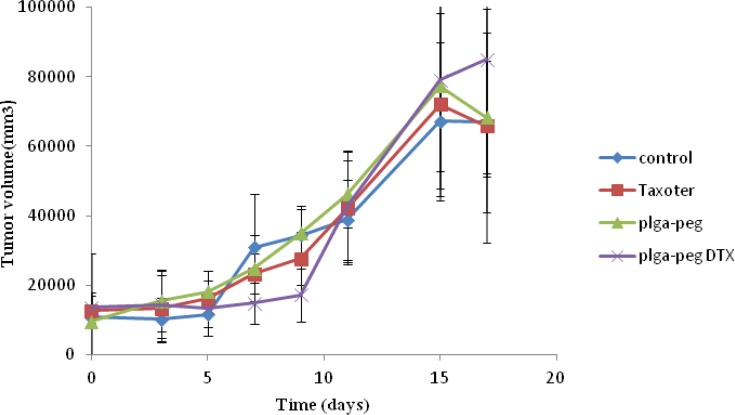
*In-vivo *antitumor effect of docetaxel loaded PEG-PLGA NPs in mice. The start day of treatment was marked as day 0. The mice were treated with 10 mg/Kg of Taxotere®, 10 mg/Kg DTX loaded NPs on days 0 respectively. Mice injected with saline were used as control groups.

Moreover, observing of body weight change undoubtedly showed that the weights of mice given docetaxel-loaded NPs and free NPs did not decrease significantly when compared to the saline control group. In contrast, equivalent dose of Taxotere® exhibited body weight lower than saline control group (Figure 6B). The survival rate of tumor-bearing mice illustrated that among mice injected with 10 mg/Kg Taxotere®, three mice died 17 days after treatment which was probably due to the toxic effects of Taxotere®. When mice were injected with 10 mg/Kg DTX NPs, demonstrated an enhanced antitumor efficacy compared to the free DTX (10 mg/Kg), six mice were alive after 17 days. The mice group treated with free NPs showed a much higher survival rate than the DTX treated group. It was considered that the tumor targeting ability of DTX NPs increased survival rates by decreasing *in-vivo *toxicity in normal tissues (32). Thus, nano-sized drug vehicles can provide advantages of reducing the high dose dependent toxicity of anticancer drugs while, at the same time increasing their anticancer efficacy. This result clearly indicate that DTX loaded NPs is comparably successful in inhibiting tumor growth while lower toxicity effect.

**Figure 6B:**
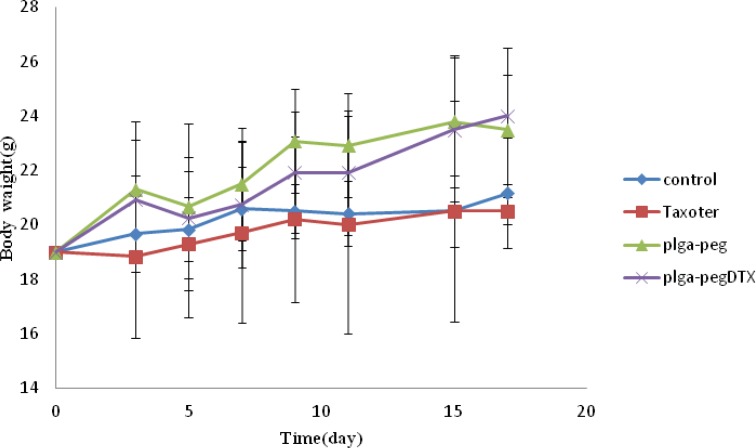
Weight changes observed in mice treated with different samples. The start day of treatment was marked as day 0. The mice were treated with 10 mg/Kg of Taxotere®, 10 mg/Kg DTX loaded NPs. Mice injected with saline and free NPs were used as control groups

**Figure 6 C:**
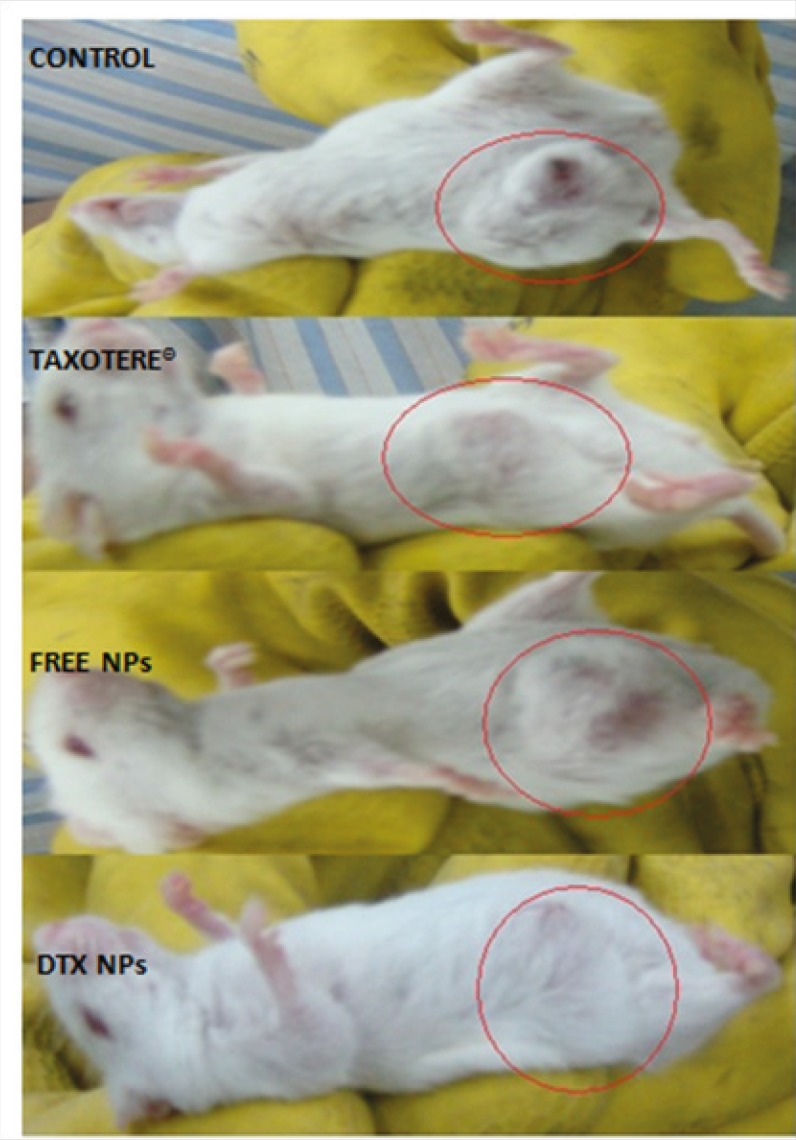
Tumor-suppressing effects five days after injection of samples. Mice in group 1 (control) received injection of saline solution. Group 2 received 10 mg/Kg Taxotere®. Group 3 were injected with NPs with no drug loading. Group 4 mice were injected with equivalent dose of 10 mg/Kg DTX-loaded NPs. Mice were treated with a single IV injection

## Conclusion

An optimized method for the preparation of DTX loaded PEG-PLGA NPs is reported in this study. Optimum values of independent variables to optimize the responses were polymer concentration of 9.75 mg/mL, drug concentration of 1.25 mg/mL, and the ratio of solvent to water of 0.31. The optimum level of size, PDI and drug loading predicted by the model were calculated to be 188 nm, 0.16 and 9% respectively. The results of *in-vitro *and *in-vivo *studies demonstrated a higher cytotoxic efficacy and less weight loss for DTX loaded PEG-PLGA NPs compared to DTX.
